# Delayed cortical development in mice with a neural specific deletion of β1 integrin

**DOI:** 10.3389/fnins.2023.1158419

**Published:** 2023-05-12

**Authors:** Mamunur Rashid, Eric C. Olson

**Affiliations:** ^1^Department of Neuroscience and Physiology, State University of New York Upstate Medical University, Syracuse, NY, United States; ^2^Department of Neurology, Columbia University Irving Medical Center, New York, NY, United States

**Keywords:** focal adhesion, paxillin, neurodevelopmental delay, cortical development, brain, β1 integrin

## Abstract

The adhesion systems employed by migrating cortical neurons are not well understood. Genetic deletion studies of focal adhesion kinase (FAK) and paxillin in mice suggested that these classical focal adhesion molecules control the morphology and speed of cortical neuron migration, but whether β1 integrins also regulate migration morphology and speed is not known. We hypothesized that a β1 integrin adhesion complex is required for proper neuronal migration and for proper cortical development. To test this, we have specifically deleted β1 integrin from postmitotic migrating and differentiating neurons by crossing conditional β1 integrin floxed mice into the *NEX-Cre* transgenic line. Similar to our prior findings with conditional paxillin deficiency, we found that both homozygous and heterozygous deletion of β1 integrin causes transient mispositioning of cortical neurons in the developing cortex when analyzed pre- and perinatally. Paxillin and β1 integrin colocalize in the migrating neurons and deletion of paxillin in the migrating neuron causes an overall reduction of the β1 integrin immunofluorescence signal and reduction in the number of activated β1 integrin puncta in the migrating neurons. These findings suggest that these molecules may form a functional complex in migrating neurons. Similarly, there was an overall reduced number of paxillin+ puncta in the β1 integrin deficient neurons, despite the normal distribution of FAK and Cx26, a connexin required for cortical migration. The double knockout of paxillin and β1 integrin produces a cortical malpositioning phenotype similar to the paxillin or β1 integrin single knockouts, as would be expected if paxillin and β1 integrin function on a common pathway. Importantly, an isolation-induced pup vocalization test showed that β1 integrin mutants produced a significantly smaller number of calls compared to their littermate controls when analyzed at postnatal day 4 (P4) and revealed a several days trend in reduced vocalization development compared to controls. The current study establishes a role for β1 integrin in cortical development and suggests that β1 integrin deficiency leads to migration and neurodevelopmental delays.

## Introduction

Appropriate adhesion to the extracellular matrix (ECM) is required for neuronal differentiation, proliferation, and migration (Raghavan et al., 2000; Du et al., 2011; Long and Huttner, 2019; Kanchanawong and Calderwood, 2022). During development of the cerebral cortex, prospective excitatory neurons exit the cell cycle and migrate across the ventricular wall to settle in their prospective layers within the developing cortical plate (CP). Migrating neurons first adopt a multipolar cellular phenotype. Multipolar neurons show a boxy cellular soma with multiple fine processes and migrate slowly through the intermediate zone (IZ). After crossing the IZ, the neuron attaches to a radial glial fiber and simultaneously adopts a bipolar morphology with a simple leading process and often a trailing axon (Nadarajah et al., 2001; Tabata and Nakajima, 2003). The neuron then migrates rapidly through the cortical plate (CP) to an area underneath the marginal zone (MZ) where the migrating neuron then begins to elaborate dendrites and develop neuronal physiological properties (Picken Bahrey and Moody, 2003; Corlew et al., 2004; O’Dell et al., 2012, 2015). The molecular mechanism underlying the different migration modes are not well understood and whether β1-integrin containing focal adhesions are essential for this process is not clear. The focal adhesion complex forms dynamic attachment points between the ECM and cytoskeleton in migratory cells including fibroblasts and some forms of metastatic cancers (Wehrle-Haller and Imhof, 2003; Kim and Wirtz, 2013; Yamaguchi and Knaut, 2022). Integrin receptors form heterodimers usually composed of an alpha and a beta subunit, combinations of which may form 24 different receptors (Takada et al., 2007; Barczyk et al., 2010), with distinct functional properties. The β1 integrin subunit can form dimers with 12 different alpha subunits, making β1 integrin receptors the most diverse class among all the integrin families (Luo et al., 2007). This diversity of receptor composition is reflected in the diversity of ECM ligands that this family of receptors can bind – among these ligands are fibronectin, laminin, and collagen (Plow et al., 2000; Lilja and Ivaska, 2018). The integrin cytoplasmic tail organizes the assembly of the hundreds of proteins that constitute the focal adhesion, a regulatory complex that connects to the actin cytoskeleton (Geiger and Zaidel-Bar, 2012; Horton et al., 2015; Dong et al., 2016; Humphries et al., 2019). Despite their importance in many cell types, the role of β1-containing integrins in neuronal migration is controversial. Early studies using engineered gene knockouts, shRNA-based knockdown and function blocking antibodies suggest that alpha 3 and alpha 5 integrins (that are believed to partner only with β1 integrin) are required for normal neuronal migration (Anton et al., 1999; Dulabon et al., 2000; Schmid et al., 2004; Marchetti et al., 2010). In contrast, genetic ablation of β1 integrin, which would be expected to eliminate both α3β1 and α5β1 functional dimer, did not disrupt radial migration (Graus-Porta et al., 2001; Belvindrah et al., 2007). In combination these results suggest either that α3 and α5 integrin have functions independent of β1 integrin or that the underlying findings are incomplete or incorrect.

While specific β1 integrin deletion from migrating neurons did not appear to disrupt the formation of the cerebral cortex assayed at postnatal day 60 (P60), deletion of β1 integrin precursors, and migrating neurons produced defects in the attachment points between the neural precursors and the basal lamina (Graus-Porta et al., 2001; Belvindrah et al., 2007). Neuronal precursors called radial glial extend processes across the entire width of the cerebral wall and attach at the pial basement membrane in elaborations called endfeet. β1 integrin deficiency caused disruption of these endfeet and associated breaches in the pial basement membrane that lead to the disrupted organization of the underlying cortical layers (Graus-Porta et al., 2001; Belvindrah et al., 2007). Previously, we showed that neural-specific deletion of paxillin, a focal adhesion adaptor protein, reduced migration speed and delayed cortical layer formation (Rashid et al., 2017). Similarly, others have shown that focal adhesion kinase (FAK) deletion delays cortical development and altered morphology of migrating neurons (Valiente et al., 2011). Thus, prior studies suggest that focal adhesion proteins have a modulatory function in maintaining the pace of neuronal migration, but whether these focal adhesion proteins are functioning together with β1 integrin is not clear.

One possible reconciliation between the findings is that β1-integrin deficiency delays cortical migration, an observation that would have been missed in the prior study that analyzed cortical structure at P60, significantly after the migration delay is resolved in FAK and paxillin-deficient mice. To test this, we utilized the *NEX-Cre* mice line, which generates Cre-dependent recombination in developing pyramidal (excitatory) neurons soon after final mitosis and prior to the period of radial migration in the cortex and hippocampus (Goebbels et al., 2006). Thus, the *NEX-Cre/*β1 conditional specifically interrogates β1 integrin function in developing excitatory neurons but leaves neural precursors (including radial glia), interneurons and glia unaffected. We then examined the formation of cortical layers in the pre- and perinatal stages for the presence of a developmental delay. We found that the phenotype of the conditional β1 integrin deletion is similar to the deletion of FAK and paxillin with abnormal neuronal positioning in the pre- and perinatal period. However, the phenotype is not apparent in the mature cortex. In addition, paxillin and β1 integrin colocalized in migrating neurons and deletion of one alters the expression of the other. Remarkably this delay of cortical formation is accompanied by a deficit in behavioral acquisition: β1 integrin deficient pups demonstrate reduced ultrasonic vocalizations (USVs) at postnatal 4 (P4) compared to littermate controls. Collectively these findings identify an important role for β1 integrin in controlling the rate of cortical development that likely involves interaction the focal adhesion proteins paxillin and FAK.

## Results

### Paxillin and β1 integrin localize in the perisomatic area and in the leading process of migrating neurons

**Figure 1:**
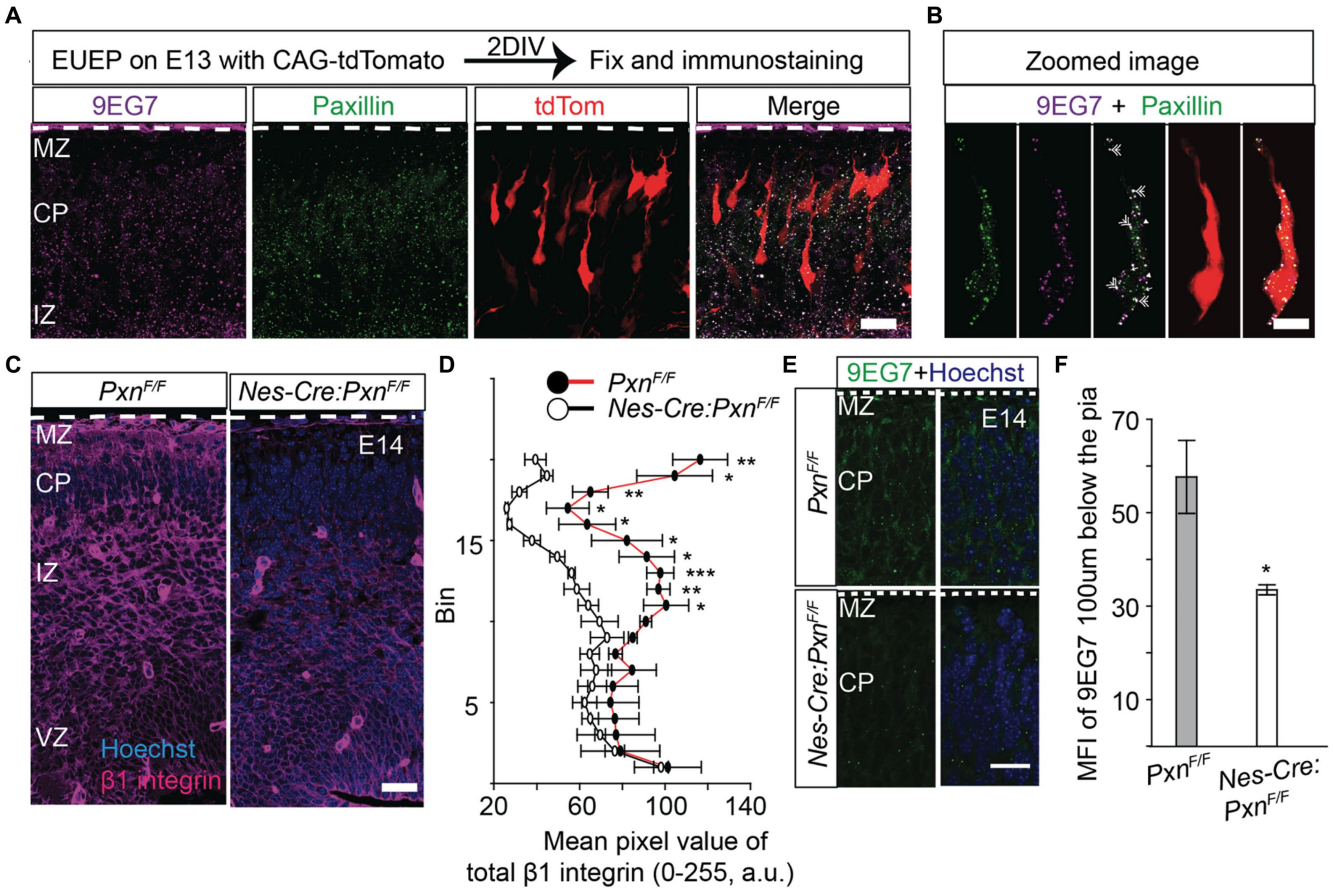
Paxillin and activated β1 integrin colocalize in the migrating neurons. To label migrating neurons embryos, were electroporated with CAG-tdTomato (red) and whole hemisphere explants were prepared. After 2 days *in vitro* (DIV) the explants were fixed and immunostained. **(A)** Representative image of a wildtype section co-stained with activated β1 integrin (magenta) and paxillin (green). **(B)** Optical zoom of a migrating neuron (red) showing overlapping pixels of 9EG7 (activated β1 integrin) and paxillin (double arrow). The neuron was digitally isolated based on the red signal to identify only those puncta associated with the cell. Also, note the single labeled paxillin (single arrow) and 9EG7 puncta (arrow head) in addition to the colocalized (white) puncta. **(C,D)** A representative E14 *Nes-Cre:Pxn^F/F^* section stained with total β1 integrin (magenta). The mean pixel intensity of β1 integrin was measured in 20 equal bins, where bin 20 is in the MZ and bin 1 is in the VZ. The mean pixel intensity in bins from 11 to 20 were significantly reduced in the *Nes-Cre:Pxn^F/F^* group (*n* = 3/group). **(E,F)** Staining of activated β1 integrin by 9EG7 (green) in the *Nes-Cre:Pxn^F/F^* shows that the mean intensity of 9EG7 was significantly reduced 100 μm below the pial border (n = 4/group). Nuclei were counter stained with Hoechst 33342 (blue). The pial surface is outlined by the dashed white line. Data were analyzed using unpaired Student’s *t*-test. *p < 0.05; *** *p* < 0.001. Scale bar: 20 μm in **(A,C,E)**; 10 μm in **(B)**.

In our previous study, we found that cell-autonomous deletion of paxillin in migrating neurons produced shorter leading process and slowed radial migration (Rashid et al., 2017). In fibroblasts and cancer cells, paxillin localizes to nascent adhesions at the leading edges of migrating cells (Digman et al., 2008; Hu et al., 2014). To determine paxillin’s localization in migrating neurons, we labeled differentiating and migrating neurons with a red fluorescence protein by electroporation of a CAG-tdTomato expression plasmid into whole hemisphere cortical explants (Nichols et al., 2013). After 2 days of development, the explants were fixed and analyzed by immunohistology. Consistent with the E14.5 *in situ* expression pattern (genepaint.org), paxillin is expressed across the cortical wall with highest levels in the ventricular zone (VZ) and intermediate zone (IZ). Within the migrating neuron the paxillin immunofluorescence signal appears as sub-resolution fluorescent puncta in both leading processes and in the perisomatic areas (Figure 1A). As paxillin is known to form a complex with β1 integrin and mediate ECM adhesion in many cell types (Chen et al., 2000; Crowe and Ohannessian, 2004), we used 9EG7, an antibody specific for activated β1 integrin, (Bazzoni et al., 1995) to determine whether active β1 integrin colocalized with paxillin migrating neurons. Activated β1 integrin was found colocalized with paxillin in puncta in the leading process and perisomatic area (Figure 1B). Consistent with this visual assessment, the Manders Correlation Coefficient indicate that 78% of the above threshold 9EG7+ pixels colocalize with above threshold paxillin pixels. The observed spatial distribution pattern is similar to the retinal neuronal cultures where paxillin co-distributed with β1 integrins subunits in the neurites (de Curtis and Malanchini, 1997).

### CNS specific deletion of paxillin alters expression of β1 integrin

Paxillin is known to coordinate cytoskeletal remodeling after the integrin receptor binds the ECM ligand. However, paxillin functions differently depending on the rigidity of the ECM. For example, when neurons are cultured on soft substrates like an embryonic brain, paxillin interacts with endocytic proteins to promote neurite outgrowth by activating the small GTPase Rac1. In contrast, when neurons are grown on stiff substrates, paxillin interacts with focal adhesion proteins, which sequester paxillin from the endocytic machinery, causing a delay in neurite outgrowth (Chang et al., 2017). This finding raises the possibility that paxillin may not be associated its traditional adhesion complex partners in the developing brain. Therefore we have looked for the status of β1 integrin in wildtype and paxillin deficient embryonic cortex (*Nes-Cre:Pxn ^F/F^*). Surprisingly, we found that the immunofluorescence signal of both activated β1 integrin and total β1 integrin were significantly lower in the paxillin-deficient group compared to the wild type control (Figures 1C–F).

### Post-mitotic deletion of β1 integrin alters the distribution of upper layer neurons at birth

**Figure 2:**
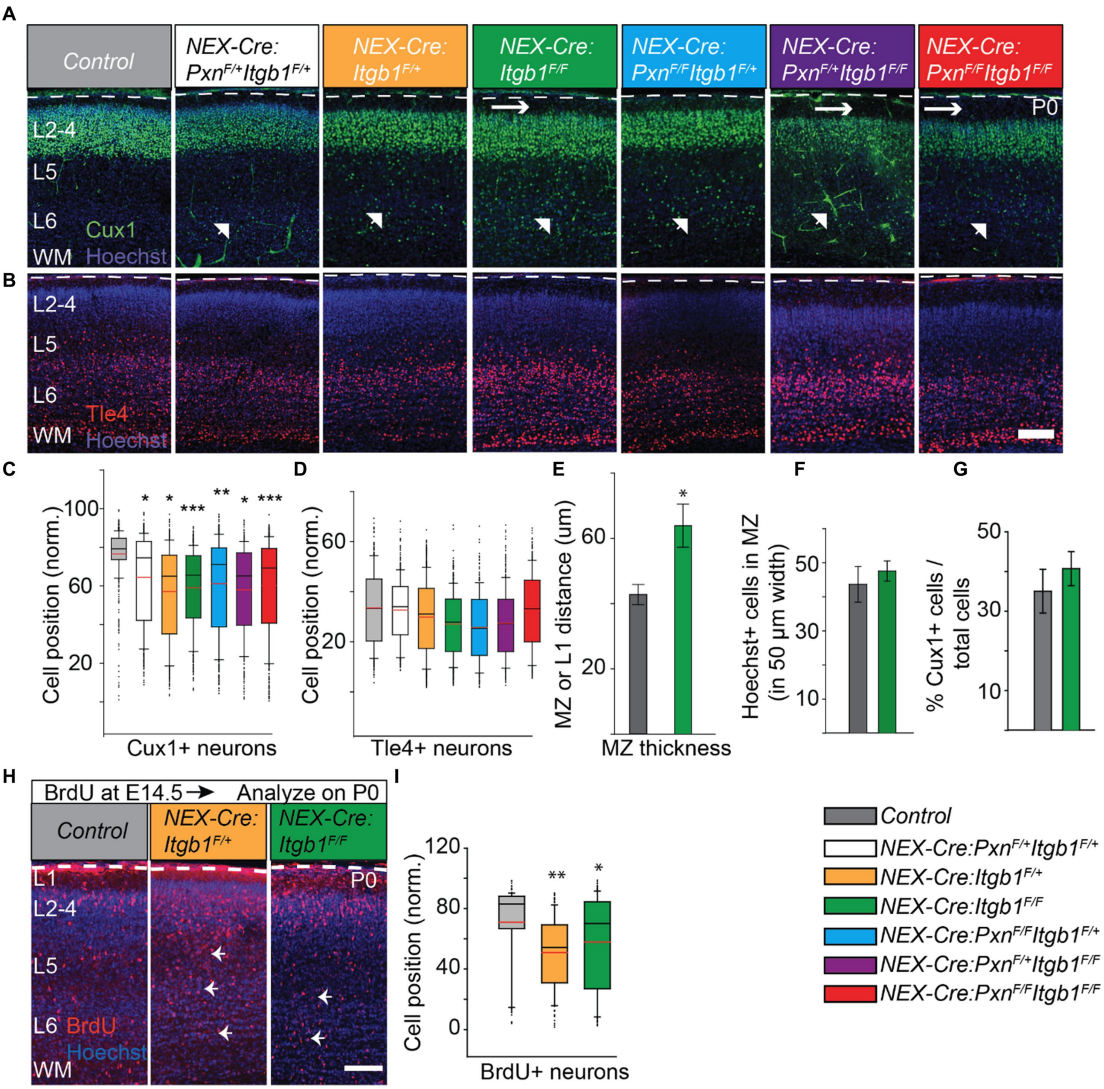
Deficiency of β1 integrin alters the distribution of upper layer neurons at P0. The positions of Cux1+ neurons that lacked β1 integrin and/or paxillin were quantified. Representative images of a control (*n* = 3); *NEX-Cre:Pxn^F/+^Itgb1^F/+^* (*n* = 3); *NEX-Cre:Itgb1^F/+^* (*n = 4*); *NEX-Cre:Itgb1^F/F^* (*n* = 4); *NEX-Cre:Pxn^F/F^Itgb1^F/+^* (*n* = 3); *NEX-Cre:Pxn^F/+^Itgb1^F/F^* (*n* = 3); *NEX-Cre:Itgb1^F/F^Pxn^F/F^* (*n* = 4) stained with **(A)** Cux1 (green) and with **(B)** Tle4 (red). β1 integrin deficiency (either heterozygous or homozygous) produced altered positioning of Cux1+ neurons (arrow head) at P0. The β1 integrin homozygous deficient group showed an enlarged MZ (horizontal arrow). **(C)** Box-and-whisker plot showed that β1 integrin deficient Cux1+ neurons were distributed broadly, and the mean position was significantly deeper compared to the controls. **(D)** The positioning of deep layer Tle4+ neurons among the group were indistinguishable at this time point. **(E)** The marginal zone of the *NEX-Cre:Itgb1^F/F^* groups (*n* = 7) was significantly thicker comparing with the control group (*n* = 6). **(F)** The number of Hoechst+ nucleii in the MZ was indistinguishable between control (*n* = 6) and the *NEX-Cre:Itgb1^F/F^* (*n* = 7) groups. **(G)** There was no difference in the percentage of Cux1+ neurons in the counting box between the groups (*n* = 3 in control, *n* = 4 in *NEX-Cre:Itgb1^F/F^*). **(H,I)** Abnormal positioning of upper layer neurons is confirmed by BrdU birth-dating experiment. **(H)** Representative images of P0 cortical sections of control, *NEX-Cre:Itgb1^F/+^*, and *NEX-Cre:Itgb1 ^F/F^* animals i.p. injected with BrdU on E15 and stained with **(H)** anti-BrdU antibodies. **(I)** Box-and-whisker plot distribution of BrdU+ neurons. The mean position of BrdU+ neurons was significantly deeper in both *NEX-Cre:Itgb1^F/+^* and *NEX-Cre:Itgb1^F/F^* compared to control (*n* = 3/group). Nuclei were counter stained with Hoechst 33342 (blue). The pial surface is outlined by the dashed white line. For multiple group comparison, the data were analyzed by using one-way ANOVA followed by *post hoc* Tukey’s test. *p < 0.05; ***p < 0.001. Scale bar: 100 μm.

Neural-specific deletion of β1 integrin did not disrupt cortical layer formation in mice examined at postnatal day 60 (Belvindrah et al., 2007). However, this prior study did not study the neuronal distribution during the migration period and would likely not have identified a migration delay like those found with conditional deletion of FAK and paxillin (Valiente et al., 2011; Rashid et al., 2017). Thus, we hypothesized that β1 integrin might have a role in neuronal migration which could be identified by pre- and perinatal analysis. As with our prior study we used layer specific markers to examine cellular distribution in the β1 integrin conditional knockout cortex. We used Cux1, a transcription factor expressed by the upper layer (layer II-IV) neurons and Tle4, a transcription factor expressed by the deep layer (layer V-VI) neurons to detect cellular distributions at different developmental time points (Nieto et al., 2004; Molyneaux et al., 2015; Rashid et al., 2017; Bartkowska et al., 2018). At postnatal day 0 (P0), we found upper layer Cux1+ cells distributed in deeper positions in both the *NEX-Cre:Itgb1^F/F^* and in the *NEX-Cre:Itgb1^F/+^* cortex. In contrast, wildtype controls were found at a more superficial position at the top of the cortical plate (CP). To understand possible genetic interaction between paxillin and integrin we have generated paxillin/β1 integrin double cKO (*NEX-Cre:Itgb1^F/F^/Pxn^F/F^*) and the resulting allelic combinations (*NEX-Cre:Pxn^F/F^Itgb1^F+^*; *NEX-Cre:Pxn^F/+^Itgb1^F+^*; *NEX-Cre:Pxn^F/+^Itgb1^FF^*). Whereas Cux1+ neurons in wildtype control cortex showed a mean position located at 75.4% ± 2.3% of the distance across the cerebral wall, the mutant genotypes showed deeper mean positions (Figures 2A,C; *NEX-Cre:Itgb1^F/+^ =* 64.5% ± 2.4%; *NEX-Cre:Itgb1^F/F^ =* 60.0% ± 2.9%; *NEX-Cre:Pxn^F/F^Itgb1^F/+^ =* 61.4% ± 1.2%; *NEX-Cre:Pxn^F/+^Itgb1^F/+^ =* 64.5% ± 0.1%; *NEX-Cre:Pxn^F/+^Itgb1^F/F^ =* 64.9% ± 0.4%; *NEX-Cre:Itgb1^F/F^Pxn^F/F^ = 59.4*% ± 1.8%; One-way ANOVA, *p* < 0.001). In contrast, there were no differences in the mean positioning of the Tle4+ deep layer cortical neurons, when analyzed at P0 (Figures 2B,D). Finally, there was no difference in the number of Cux1+ cells in the entire counting area between control and β1 integrin deficient groups (Figure 2G) suggesting that the altered distribution is not due to altered neuronal production. Together the findings show that paxillin and β1 integrin deficiency produces similar migration phenotype, with an abnormal distribution of Cux1+ neurons at P0. Importantly, the β1 integrins heterozygous (*NEX-Cre:Itgb1^F/+^*) alone produces a distribution of cells similar to the paxillin deletion phenotype. This may be mechanistically significant as deletion of paxillin causes ~50% reduction of total β1 integrin immunofluorescence in the cortical plate (Figures 1C,D). However western blot analyses of total cortical lysates derived from embryonic hemispheres deficient in paxillin and the paxillin family member Hic-5 revealed wildtype levels of β1 integrin protein (Supplementary Figure S4). Thus, the altered β1 integrin IHC signal observed with paxillin deficiency may reflect altered the distribution or conformational state of β1 integrin.

Conditional deletion of β1 integrin in neurons caused an additional phenotype which was not observed with conditional paxillin deficiency. We found that the thickness of the MZ layer of β1 integrin deficient cortices was 48% larger at P0 compared to control. In principle, this could be due to an increased number of cells but a count of Hoechst+ nuclei along the MZ suggested that the absolute cell number in the MZ was not different in the mutant (Figures 2E,F). This would seem to suggest that β1 integrin deficiency increases ECM deposition, increases cell volume, or increases neurite growth in the mutant, possibilities that require further examination.

### BrdU birth-dating confirms the altered distribution of upper layer neurons at birth

To further confirm the altered distribution identified by Cux1 immunolabeling, we introduced the nucleoside analog BrdU (bromodeoxyuridine) to the E15 pregnant mice to birthdate neurons fated for upper cortical layers (Takahashi et al., 1999). Upon analyses at P0, we found that the mean positions of BrdU+ neurons were also significantly deeper in both *NEX-Cre:Itgb1^F/+^* and *NEX-Cre:Itgb1^F/F^* cortex compare to littermate controls (Figures 2H,I; control = 74.0% ± 3.0%; *NEX-Cre:Itgb1^F/+^ =* 51.0% ± 2.9%; *NEX-Cre:Itgb1^F/F^ =* 57.0% ± 2.4%; one-way ANOVA, *p* < 0.01).

### Normal positioning of upper layer neurons at postnatal day 35

**Figure 3:**
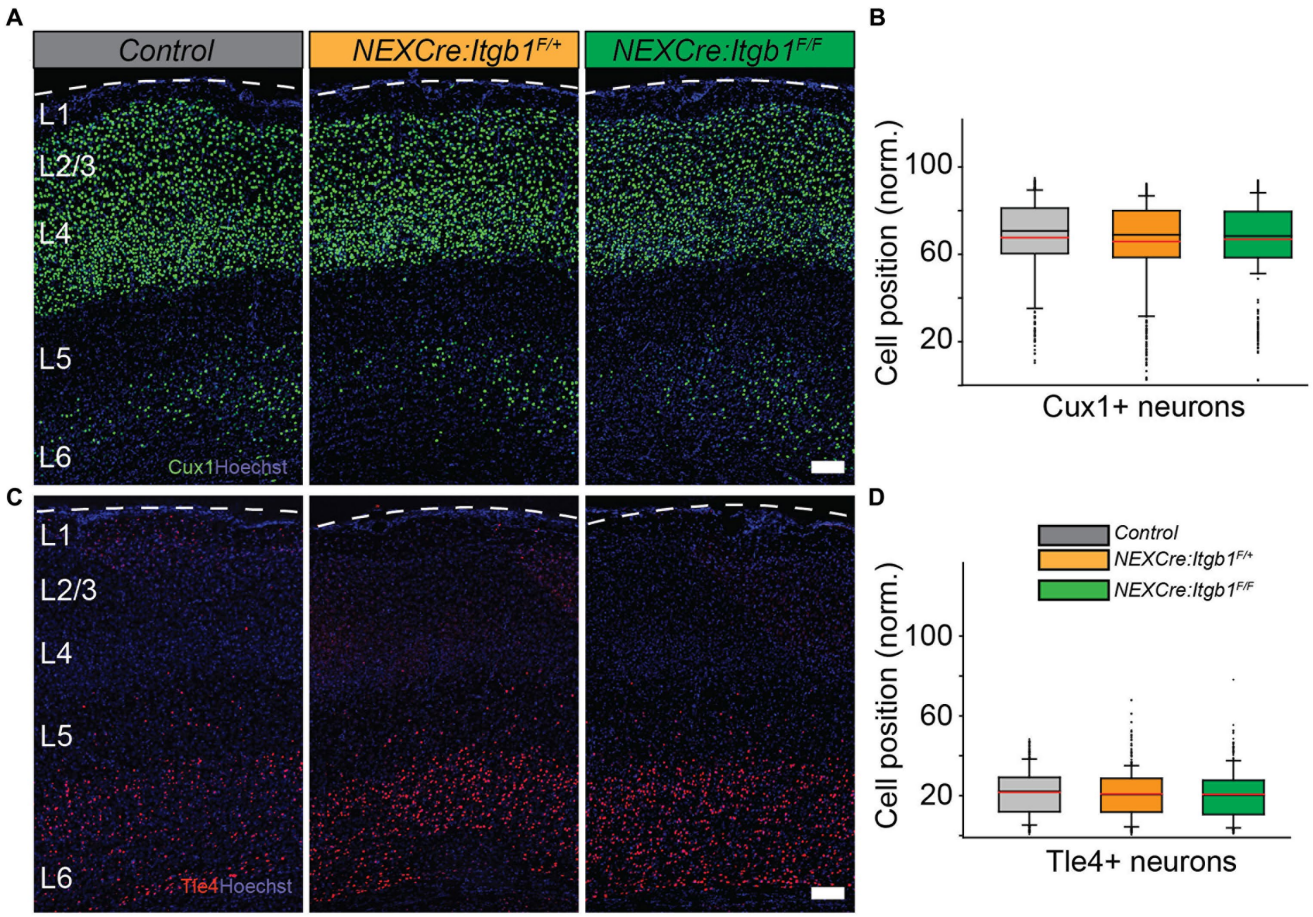
Normal distribution of upper layer neurons in β1 integrin deficient cortex at P35. Representative images of cortical sections from control (*n* = 4), *NEX-Cre:Itgb1*^*F/*+^(*n* = 5), and *NEX-Cre:Itgb1^F/F^* (*n* = 4) stained with **(A)** anti Cux1 and **(C)** anti Tle4 antibodies. **(B)** Box-and-whisker plot distribution of Cux1+ neurons. The mean position of Cux1+ neurons among the groups were indistinguishable. **(D)** Box-and-whisker plot distribution of Tle4+ neurons. The mean position of Tle4+ neurons among the groups were indistinguishable. Nuclei were counter stained with Hoechst 33342 (blue). The pial surface is outlined by dashed white line. Data were analyzed by using one-way ANOVA followed by *post hoc* Tukey’s test. Scale bars: 100 μm in **(A,C)**.

Previously, it was reported that *NEX-Cre* mediated deletion of β1 integrin does not display any cortical migration phenotype when analyzed at P60. To test whether the migration phenotype persists in the mature cortex, we measured Cux1 and Tle4 positioning at P35. Consistent with the prior findings we did not find any positioning abnormalities in both upper or deep layer neurons in neither *NEX-Cre:Itgb1^F/+^* nor in *NEX-Cre:Itgb1^F/F^* when compared to control groups (Belvindrah et al., 2007; Figures 3A–D). For Cux1: control = 67.8% ± 1.3%; *NEX-Cre:Itgb1^F/+^ =* 65.8% ± 1.1%; *NEX-Cre:Itgb1^F/F^ =* 66.9% ± 0.5%. For Tle4: control = 21.5% ± 1.3%; *NEX-Cre:Itgb1^F/+^ =* 20.7% ± 0.7%; *NEX-Cre:Itgb1^F/F^ =* 20.5% ± 1.0%; *p* > 0.05. This result demonstrates that the migration phenotype due to neuronal deletion of β1 integrin is transient and is similar to the paxillin deletion phenotype (Rashid et al., 2017).

### Altered position of deep cortical layer neurons at E14.5 and E16

**Figure 4:**
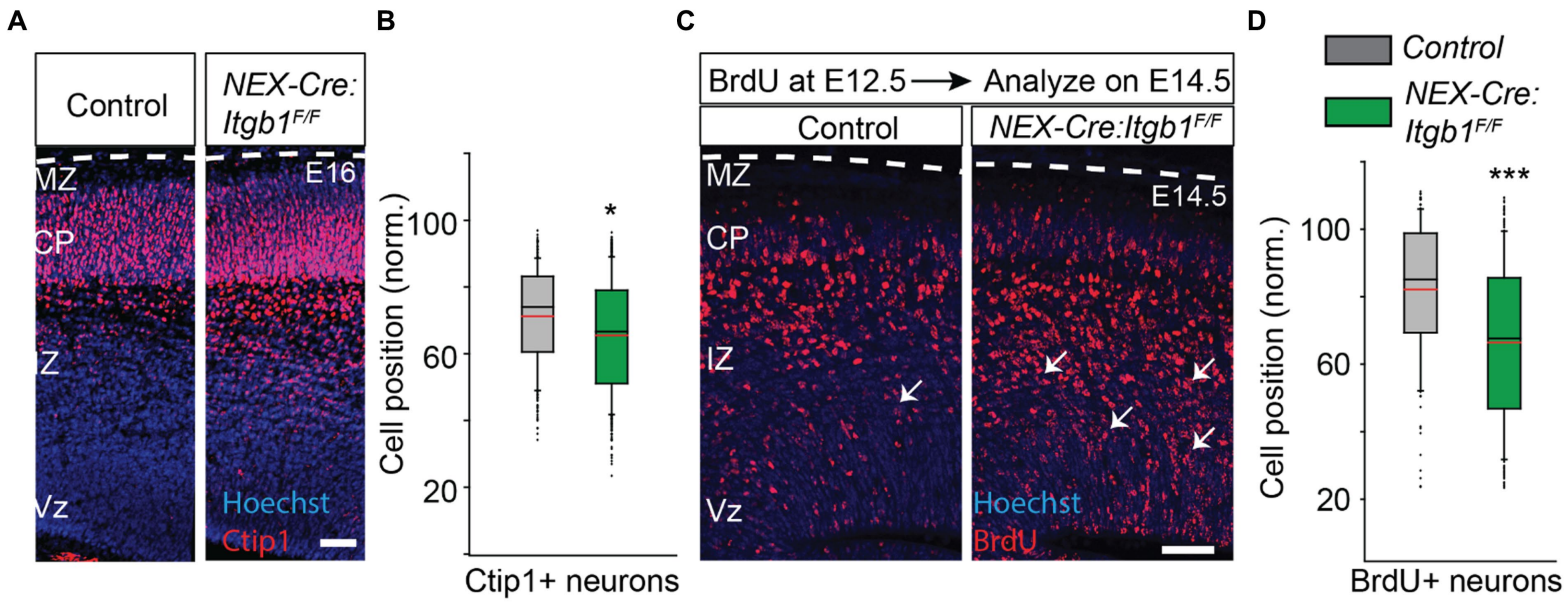
Abnormal positioning of deep layer neurons is revealed when analyzed prenatally. **(A)** Representative image of E16 coronal sections stained with Ctip1 (red). **(B)** The positioning of Ctip1+ neurons in the *NEX-Cre:Itgb1^F/F^* was significantly deeper in the mutant cortex (*n* = 3/group). **(C)** Representative images of E14.5 cortical sections of control and *NEX-Cre:Itgb1^F/F^* animal i.p. injected with BrdU on E12.5 and immunostained with anti-BrdU antibodies (red). **(D)** The mean position of BrdU + neurons was significantly deeper in the mutant group (*n* = 3 in control, *n* = 4 in mutant). Nuclei were counter stained with Hoechst 33342 (blue). The pial surface is outlined by dashed white line. Data were analyzed by using unpaired Student’s *t*-test. ***p < 0.001. Scale bar: 50 μm.

To identify potential deep layer neuronal positioning defects, we need to analyze earlier time points than P0, as deep layer migration is largely complete by mid-corticogenesis (E16). In addition, deep layer neurons do not migrate as far compared to the upper layer neurons, so the absolute difference in mean distribution between integrin-deficient and control groups would be expected to be smaller. Therefore we used the neuronal marker Ctip1 to identify the migrating neurons, which initiates expression in the upper intermediate zone and persists in the cortical plate (Rashid et al., 2017). While Ctip1 is not a specific marker of deep layer neurons in the adult cortex, at this early time point the CP is composed almost exclusively of deep layer excitatory neurons. Thus, the distribution of the post mitotic marker Ctip1 largely reflects the distribution of deep cortical layer cells. We found that the position of Ctip1+ neurons was 8% deeper in β1 integrin mutant when analyzed on E16 (Figures 4A,B) (control = 71.4% ± 1.3%; *NEX-Cre:Itgb1^F/F^ =* 65.5% ± 0.5%; *p* < 0.05), which suggests that deep layer migration is also disrupted by β1 integrin deficiency. To further confirm this finding, we performed a birth-dating experiment by IP injecting BrdU on E12.5 (to target deep layer cells) (Takahashi et al., 1999) followed by analysis on E14.5. In accordance with the Ctip1+ findings, we found that the mean position of BrdU+ neurons was significantly deeper in the β1 integrin deficient cortex (Figures 4C,D; control = 62.0%; ± 1.4%; *NEX-Cre:Itgb1^F/F^ =* 46.0%; ± 0.6%; *p* < 0.001). These results suggest that like paxillin deletion (Rashid et al., 2017), deletion of β1 integrin also causes a positioning defect of both upper and deep layer neurons.

### Reduced number of activated β1 integrin puncta in the absence of paxillin

**Figure 5:**
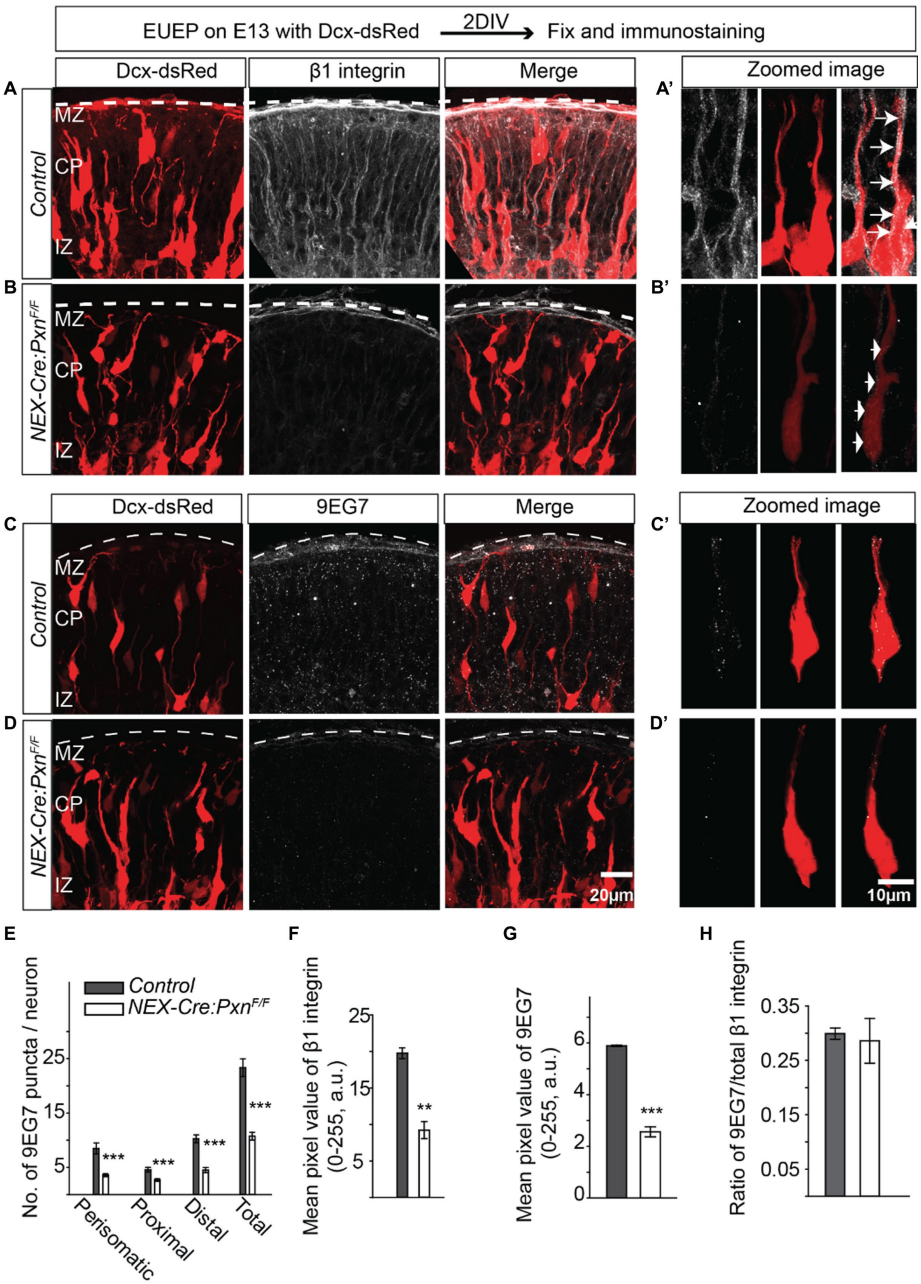
Reduced number of β1 integrin puncta in the paxillin-deficient neurons. **(A-A’)** Schematic of the experimental design. A representative image of control sections stained with total β1 integrin (white). An optically zoomed image of a wildtype migrating neuron showing expression of β1 integrin along the leading process and perisomatic areas (arrows). **(B-B′)** A representative image of *NEX-Cre:Pxn^F/F^* sections stained with total β1 integrin (white). An optically zoomed image of migrating neurons showing fewer β1 integrin puncta along the leading process and perisomatic areas (arrows) in the absence of paxillin. **(C-C′)** A representative image of control sections stained with activated β1 integrin (9EG7) (white). An optically zoomed image of migrating neurons showing expression of 9EG7 along the leading process and perisomatic areas. **(D-D′)** A representative image of *NEX-Cre:Pxn^F/F^* sections stained with 9EG7 (white). An optically zoomed image of migrating neurons shows an overall lower number of activated β1 integrin along the leading process and peri somatic areas in the absence of paxillin. **(E)** Quantification of the number of 9EG7 puncta in different areas of the migrating neurons. The number of 9EG7 puncta was significantly lower in all areas across perisomatic, proximal, and distal leading process (*n* = 12 cells/group). **(E–H)** The mean pixel value of total β1 integrin and activated β1 integrin 70um below pial surface were significantly reduced in the paxillin mutant group (*n* = 3/group). There is no difference in the ratio of activated vs. total β1 integrin. The pial surface was outlined by a dashed white line. Data were analyzed by using unpaired Student’s *t*-test. Scale bar: 20 μm for **(D)**; 10 μm for **(D′)**. **p < 0.01, ***p < 0.001.

The overlapping staining pattern of paxillin and activated β1 integrin in the migrating neurons and the decreased β1 integrin expression in the paxillin deficient (*Nestin-Cre*) cortex (Figure 1) would be expected if the two proteins interact in migrating neurons. To formally test whether deletion of paxillin in postmitotic neurons also causes a similar dysregulation of β1 integrin expression, we fluorescently labeled neurons in *NEX-Cre:Pxn^F/F^* embryos by introducing a Dcx-dsRed expression plasmid via *ex utero* electroporation (EUEP). Dcx-dsRed is expressed by postmitotic neurons and therefore labels only migrating neurons and not the radial glial cells (Francis et al., 1999; Wang et al., 2007). The expression pattern of total β1 integrin was significantly lower in the cortical plate of *NEX-Cre:Pxn^F/F^* group (Figures 5A,B,F; control = 19.7 ± 0.7; *NEX-Cre:Pxn^F/F^ = 9.2* ± 1.2; *p* < 0.01). The activated integrin (9EG7+) puncta were distributed along the whole neuron including the perisomatic area, proximal leading process and in distal leading process in control while the *NEX-Cre:Itgb1^F/F^* sections little immunoreactivity for either 9EG7 or total β1 integrin in the migrating neuron (asterisk; Supplementary Figure S1). However, there was immunoreactivity which appears to be associated with radial glia precursor cells (arrow) that would not be targeted by *NEX-Cre* mediated deletion (Supplementary Figure S1). The no primary control revealed no significant signal in both WT and β1 integrin KO (*NEX-Cre:Itgb1^F/F^*; Supplementary Figure S1). The number of integrin+ puncta in all the regions of the paxillin deficient neuron was ~50% lower compared to controls (Figure 5E; perisomatic area: control = 8.5 ± 1.0; *NEX-Cre:Pxn^F/F^* = 3.6 ± 0.3; *p* < 0.001, proximal leading process: control = 4.5 ± 0.4; *NEX-Cre:Pxn^F/F^* = 2.7 ± 0.2; p < 0.001, distal leading process: control = 10.3 ± 0.7; *NEX-Cre:Pxn^F/F^* = 4.5 ± 0.5; *p* < 0.001). The overall mean intensity of activated β1 integrin signal was significantly reduced in the CP of the paxillin mutant group (Figures 5C,D,G; control = 5.9 ± 0.03; *NEX-Cre:Pxn^F/F^* = 2.6 ± 0.2; p < 0.001). However, similar to the *Nestin-Cre* specific paxillin deletion (Figures 1C–F), the ratio of activated vs. total β1 integrin intensity was unaffected in the *NEX-Cre* specific paxillin mutant group (Figure 5H; control = 0.3 ± 0.01; *NEX-Cre:Pxn^F/F^* = 0.3 ± 0.04; *p* > 0.05). To test whether paxillin deficiency disrupts the expression of other adhesion molecules we examined N-cadherin expression for control, *NEX-Cre:Pxn^F/F^* and *NEX-Cre:Itgb1^F/F^* groups. The expression pattern of N-cadherin was unaltered among the groups suggesting that paxillin deficiency does not cause a global alteration of adhesion protein expression (Supplementary Figure S2; control = 0.3 ± 0.01; *NEX-Cre:Pxn^F/F^* = 42.2 ± 2.2; *NEX-Cre:Itgb1^F/F^* = 42.2 ± 2.0; *p* > 0.05). These results confirm at the level of the migrating neuron, that paxillin deficiency leads to a selective decrease in both total and activated β1 integrin immunosignal.

### Fewer paxillin positive puncta in the absence of β1 integrin

**Figure 6:**
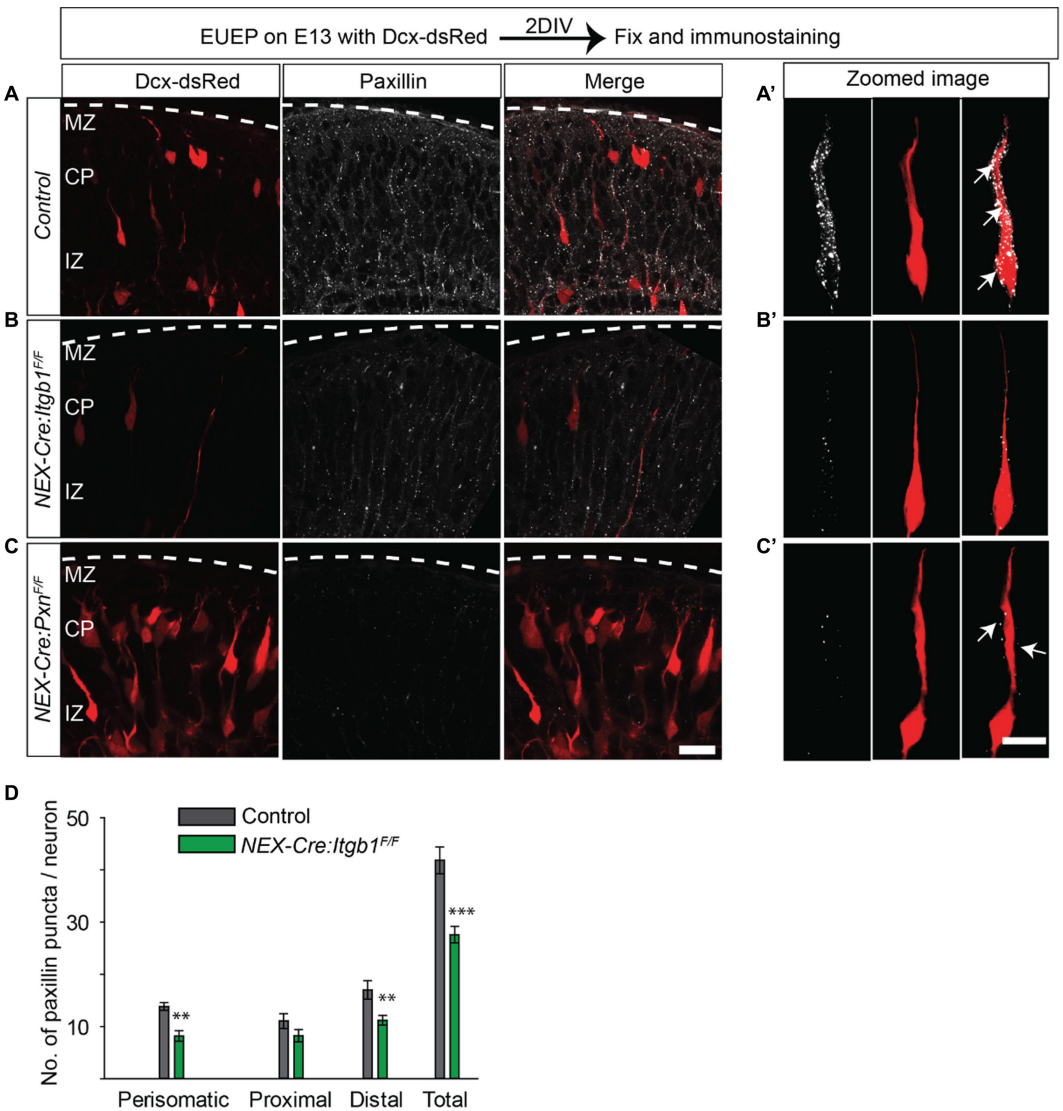
Fewer paxillin puncta in the β1 integrin-deficient neurons. **(A-A’)** Schematic of the experimental design. A representative image of paxillin (white) expression in a control section. An optically zoomed image of migrating neurons showing expression of paxillin puncta along the leading process and perisomatic areas (arrows). **(B-B′)** A representative image of *NEX-Cre:Itgb1^F/F^* sections immunostained with paxillin (white). An optically zoomed image of migrating neurons showing reduced expression of paxillin along the leading process and perisomatic areas (arrows) in the absence of β1 integrin. **(C-C′)** A representative image of paxillin knockout control sections (*NEX-Cre:Pxn^F/F^)* immunostained with anti-paxillin antibody (white). An optically zoomed image of a migrating neuron. **(D)** Quantification of the number of paxillin puncta in different areas of the migrating neurons. The number of paxillin puncta was significantly lower in perisomatic and in distal leading process (*n* = 6 cells for control, *n* = 10 cells for *NEX-Cre:Itgb1^F/F^* group). Pial surface was outlined by dashed white line. Data were analyzed by using unpaired Student’s *t*-test. Scale bar: 20 μm for C; 10 μm for **(C′)**. **p < 0.01, ***p < 0.001.

We next asked if paxillin expression was disrupted in the absence of β1 integrin. We found the total number of paxillin+ puncta were significantly reduced in the *NEX-Cre:Itgb1^F/F^* group compared to the control. The total number of puncta was 34% lower in the mutants compared to control. The deficiency in paxillin puncta was most pronounced in the perisomatic area and distal leading process (Figures 6A–D; perisomatic area: control = 13.8 ± 0.7; *NEX-Cre:Itgb1^F/F^* = 8.2 ± 1.0; *p* < 0.01, proximal leading process: control = 11.0 ± 1.4; *NEX-Cre:Itgb1^F/F^* = 8.2 ± 1.2; *p* > 0.05), distal leading process: control = 17 ± 7.8; *NEX-Cre:Itgb1^F/F^* = 11.2 ± 0.9; *p* < 0.01, total: control = 41.8 ± 2.5; *NEX-Cre:Itgb1^F/F^* = 27.6 ± 1.6; *p* < 0.001). This result suggests some form of reciprocal regulation between β1 integrin and paxillin in the establishment or maintenance of puncta in the migrating neurons.

### Normal expression pattern of FAK and Cx26 in both paxillin and β1 integrin deficient neurons

Focal adhesion kinase (FAK) and connexin 26 (Cx26) were previously reported to colocalize in puncta and regulate glial-guided migration (Elias et al., 2007; Valiente et al., 2011). In this prior study, deficiency in FAK cell-autonomously altered migrating neuron morphology and delayed migration, likely by disrupting Cx26 containing adhesions along the radial glial fiber. Importantly, a form of FAK that lacked a paxillin interacting domain could not rescue the migration defect caused by FAK deficiency. Thus, we examined the expression pattern of FAK among control, paxillin mutant, and β1 integrin mutants. Surprisingly, we did not find any difference among the groups (Supplementary Figures S3D–F,H; control = 18.7 ± 0.1; *NEX-Cre:Pxn^F/F^* = 16.9 ± 1.4; *NEX-Cre:Itgb1^F/F^* = 19.4 ± 0.3; *p* > 0.05). However, the expression pattern FAK was unusual in that the antibody recognized antigen in both in the cytoplasm and in the nucleus, which is unlike the expression pattern of paxillin and β1 integrin. While the nuclear expression (Supplementary Figures S3D–F) of FAK may be artifactual, FAK is reported to shuttle between nucleus and focal adhesion sites (Serrels et al., 2015; Sun et al., 2018). Similarly, we did not detect a significant difference in the expression pattern of Cx26 between mutants and control, although the distal leading process of the β1 integrin knockout shows a trend towards more Cx26+ puncta (Supplementary Figures S3A–C,G; perisomatic area: control = 5.6 ± 0.2; *NEX-Cre:Pxn^F/F^* = 5.9 ± 0.4; *NEX-Cre:Itgb1^F/F^* = 5.6 ± 0.6; *p* > 0.05, proximal leading process: control = 2.3 ± 0.2; *NEX-Cre:Pxn^F/F^* = 2.6 ± 0.3; *NEX-Cre:Itgb1^F/F^* = 2.8 ± 0.1; *p* > 0.05, distal leading process: control = 3.8 ± 0.2; *NEX-Cre:Pxn^F/F^* = 3.7 ± 0.3; *NEX-Cre:Itgb1^F/F^* = 4.6 ± 0.2; *p* = 0.045). Total control = 11.7 ± 0.4; *NEX-Cre:Pxn^F/F^* = 12.3 ± 0.6; *NEX-Cre:Itgb1^F/F^* = 13.0 ± 0.7; *p* > 0.05). Thus, despite the overlapping cellular phenotypes among FAK, paxillin, and β1 integrin deficient neurons, we did not find dysregulation of FAK or Cx26 expression in the β1 integrin or paxillin mutants.

### β1 integrin deficient pups produce fewer ultrasonic vocalizations

**Figure 7:**
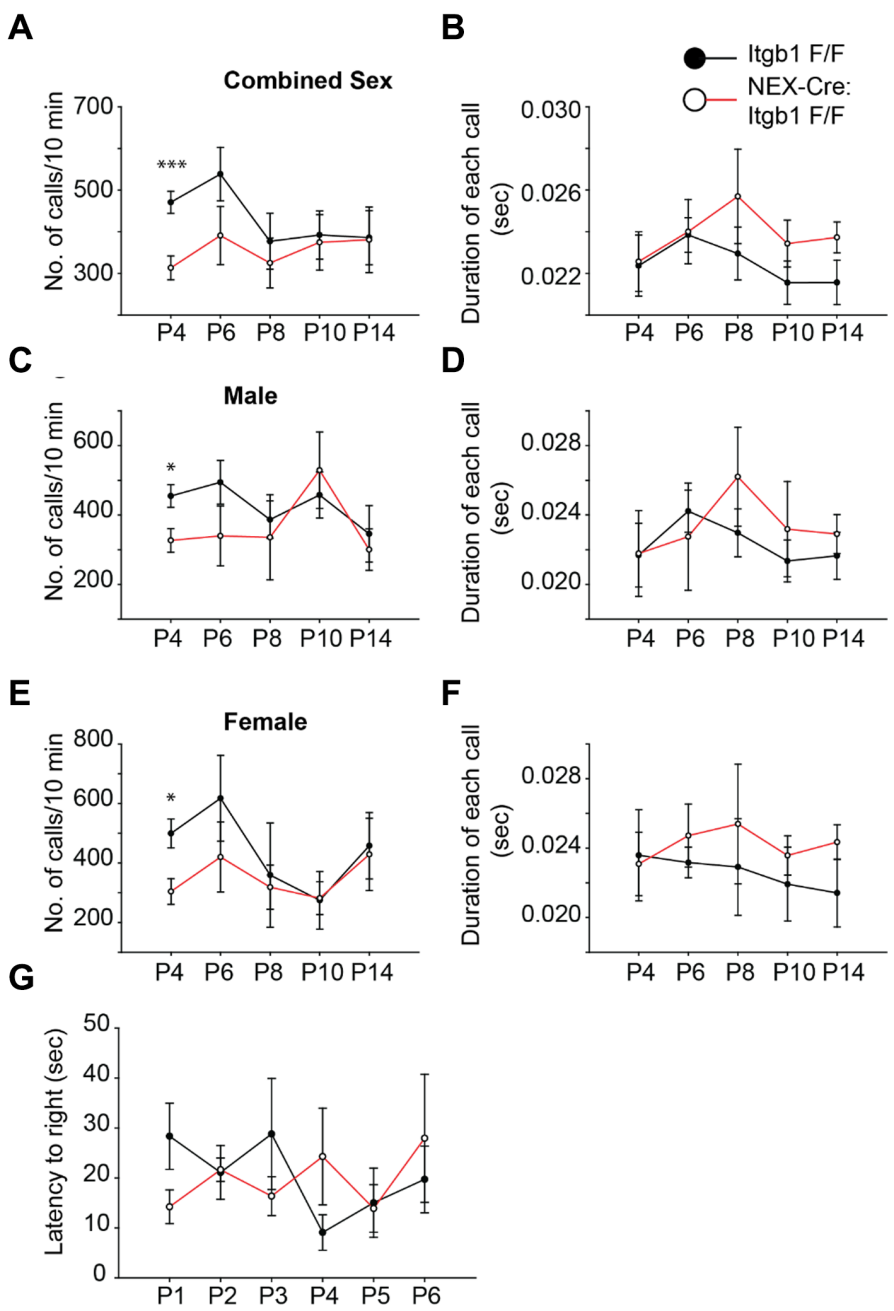
β1 integrin deficient pups show a reduction in USV call at P4. **(A,C,E)** The numbers of USVs calls/10 min were significantly reduced in the *NEX-Cre:Itgb1^F/F^* pups compared to littermate controls (*Itgb1^F/F^*) at P4. The effect was found with both genders (*n* = 14 in control, n = 15 in *NEX-Cre:Itgb1^F/F^*); male (*n* = 9 in control, n = 6 in *NEX-Cre:Itgb1^F/F^*), female (*n* = 5 in control, *n* = 9 *NEX-Cre:Itgb1^F/F^*). However there was no significant difference at P6 (*n* = 14 in control, *n* = 11 in *NEX-Cre:Itgb1^F/F^*), P8, P10, and P14 (*n* = 14 in control, *n* = 8 in *NEX-Cre:Itgb1^F/F^*). **(B,D,F)** The duration of each call was measured and compared between littermate control and *NEX-Cre:Itgb1^F/F^* pups. There was no difference in the duration of the calls at any time point. **(G)** There was no difference between the groups in the righting time for the pups at P1 (*n* = 9 in control, *n* = 5 in *NEX-Cre:Itgb1^F/F^*), P2 (*n* = 7 in control, *n* = 5 in *NEX-Cre:Itgb1^F/F^*), P3 (*n* = 5 in control, *n* = 3 in *NEX-Cre:Itgb1^F/F^*), P4 (*n* = 7 in control, *n* = 5 in *NEX-Cre:Itgb1^F/F^*), P5 (*n* = 5 in control, *n* = 3 in *NEX-Cre:Itgb1^F/F^*), and P6 (*n* = 5 in control, *n* = 3 in *NEX-Cre:Itgb1^F/F^*). Data were analyzed by using unpaired Student’s *t*-test. *p < 0.05, ***p < 0.001.

The delay of cortical development in the absence of focal adhesion proteins raises the possibility of functional delay in appropriate behavioral development. Neonatal mouse pups emit ultrasonic vocalizations (USVs) when they are isolated from the dam and littermates. Importantly, this behavior has a characteristic developmental profile with both the number of calls and the duration of calls peaking during the first two postnatal weeks (Hofer et al., 2002). Therefore, we hypothesized that this developmental profile would be delayed in pups lacking β1 integrin. We used an automated system (Avisoft) to record USVs from pups isolated on successive days between P4 and P14. Compared to controls *(Itgb1^F/F^),* the number of USVs were reduced by 33% in the β1 integrin mutant group (*NEX-Cre:Itgb1^F/F^*) at P4, the first time point analyzed (Figure 7A) (Itgb1^*F/F*^ = 470.7 ± 26.6; *NEX-Cre:Itgb1^F/F^* = 313.2 ± 28.5; *p* < 0.001). Interestingly, the difference between the groups diminished during the following 10 days. Compared to littermate controls, the β1 integrin mutant showed 27, 14, 4, and 1% reduction in call number at P6, P8, P10, and P14, respectively. The differences appear to be independent of sex (Figures 7C,E). In contrast, the duration of calls were indistinguishable between the group (Figures 7B,D,F). In addition, we performed a righting reflex test on the β1 integrin deficient pups and compared them with the littermate controls but found no difference in the latency of righting (Figure 7G). This suggests that general locomotor abilities and body strength is not affected in the mutant pups. The USVs provides preliminary evidence that the delay in brain development caused by specific deletion of β1 integrin in the forebrain causes a disruption in behavioral development.

## Discussion

In this study, we demonstrated that neuronal-specific deletion of β1 integrin caused neuronal migration delay, cortical neuronal mispositioning, and reduced number of ultrasonic vocalizations. At a molecular level, we have shown that β1 integrin colocalized with known focal adhesion adaptor protein paxillin, that deletion of paxillin alters the number of β1 integrin puncta in the migrating neurons and vice versa.

Similar to the Belvindrah et al. study, we did not observe any abnormalities in the cellular layering of adult *NEX-Cre:Itgb1^F/F^* cortex (Belvindrah et al., 2007). In the current study, we have found that β1 integrin is cell-autonomously required for appropriate cell positioning in the prenatal and perinatal period. The major difference between the studies are the time points of analyses. In our study, we looked for potential migration delay at prenatal and perinatal stages like that observed with FAK and paxillin deletion (Valiente et al., 2011; Rashid et al., 2017). We confirmed the migration phenotype by using BrdU birthdating. While the integrin floxed mice target different exons, exon 1 (Graus-Porta et al., 2001) vs. exon 3 (Raghavan et al., 2000), both delete β1 integrin completely upon Cre recombinase introduction making the results comparable.

Our findings are consistent with reports that suggest focal adhesion proteins can contribute to migration disruptions. For example, C3G-Rap1 mediated inside-out activation of α5β1 integrin is required for neuronal migration (Sekine et al., 2012; Shah et al., 2016). However, a more recent study has indicated that that Zeb2-mediated downregulation of β1 integrin surface expression may enable timely migration from the subventricular zone (Epifanova et al., 2021). Similarly, elevated expression of β1 integrin and FAK in glia due to deletion of MARVELD1, a novel nuclear protein, also caused aberrant neuronal migration (Liu et al., 2018). In conjunction with the present study, the findings suggest that β1 integrin protein is required for normal migration, but also that dynamic regulation of functional β1 integrin occurs as neurons migrate across the cerebral wall.

Besides the migration phenotype, we also observed expanded MZ in the lateral cortex of the β1 integrin mutant when analyzed at P0. The reason for expanded MZ is not clear but it is possible that ECM composition in the MZ may be altered as the total number of cells within the MZ remains unchanged (Figure 2H). Different classes of ECM are reported to be remodeled by β1 integrin (Larsen et al., 2006; Shi and Sottile, 2008). Whether β1 integrin deletion alters expression of MZ matrix proteins like members of the chondroitin sulfate proteoglycan family (CSPGs) or fibronectin has yet to be determined.

Our study suggests that paxillin and β1 integrin work together to control the speed of neuronal migration. Immunofluorescence analysis showed that deletion of paxillin causes an ~50% reduction of total β1 integrin immunohistology signal and secondly, that the β1 integrin heterozygote (*NEX-Cre:itgb1^F/+^*) that presumably expresses ~50% of normal β1 integrin protein levels also shows the neuronal mispositioning phenotype. These observations outline a model in which paxillin is required for normal integrin functional expression and deficiency in integrin functional expression slows the rate of radial migration. The decrease of β1 integrin immunofluorescence expression in migrating neurons might suggest that paxillin-deficiency destabilizes β1 integrin at the cell surface and increases the rate of endocytosis and lysosomal degradation. Paxillin has been found to control the endocytosis of β1 integrin in the brain (Chang et al., 2017) and may also control inside-out integrin activation through kindlin-2 and talin (Montanez et al., 2008; Malinin et al., 2009; Theodosiou et al., 2016). Our results also show that the overall number of paxillin puncta was also affected in migrating neurons lacking β1 integrin (Figure 6). Whether the size of these puncta was also affected could not be determined due to the resolution limit of the confocal microscope. The overall reduced number of puncta suggests that adhesion mediated by the β1 integrin/paxillin complex is required for proper neuronal migration.

Although we do not know the exact ECM molecules which interact with β1 integrins during cortical neuronal migration, the developing cerebral cortex is known to express different ECM molecules including fibronectin and laminin (Pearlman and Sheppard, 1996; Franco and Muller, 2011; Long and Huttner, 2019). During the development of the chicken optic tectum, radial glia produce fibronectin and the localization of fibronectin is aligned along the radial fibers during radial migration (Stettler and Galileo, 2004). The global knockout of fibronectin, β1 integrin, and paxillin are embryonic lethal and have shared phenotype suggesting the possible signaling axis exist among these molecules (George et al., 1993; Yang et al., 1993; Fassler and Meyer, 1995; Stephens et al., 1995; Hagel et al., 2002). This raises the possibility that β1 integrin in the migrating neurons binds to fibronectin while migrating along the radial glia (Sheppard et al., 1995; Stettler and Galileo, 2004) and this interaction is important to maintain the pace of neuronal migration. Although it is surprising that neurons still migrate in the absence of β1 integrin, the other adhesion system like Cx26 mediated adhesion (Elias and Kriegstein, 2008) remain largely unaltered thus allowing neurons to migrate.

We did not find any difference in the expression pattern of FAK or Cx26 in the paxillin or in β1 integrin mutant groups (Supplementary Figure S3). The immune reactivity of FAK was found in the nucleus and in cytoplasmic puncta, independent of genotypes. This could suggest that FAK may shuttle between adhesion sites and nucleus as has been reported in other cell types (Lim, 2013). To the best of our knowledge, nuclear localization of FAK in migrating neurons has not been reported, however, nuclear FAK was previously identified to exert its anti-tumor activity by regulating transcription of cytokines and chemokines in cancer cells (Serrels et al., 2015). In addition, FAK has nuclear localization sequences (NLS) (Lim et al., 2008) that might explain its transport to the nucleus, but the exact role of nuclear FAK has yet to be resolved.

The observation of a period of reduced USVs in the β1 integrin deficient neonatal pups is novel and could indicate a broader neurodevelopmental delay. Cortical neuronal positioning and migration defects have previously been associated with altered ultrasonic vocalization in mouse pups. For example, deletion of *Foxp1*, a transcription factor implemented in neurodevelopment, by using the *Emx1-Cre* line, causes abnormal Cux1 neuronal positioning in the deep cortex at P7 and produces a lower number of whistle call when analyzed at P4 (Usui et al., 2017). Similarly, *Cntnap2* knockout mice exhibits abnormal Cux1 positioning and lower USVs (Penagarikano et al., 2011). Cortico-striatal circuits are commonly implicated in vocal communication of different species like mice, songbirds, and human (Murugan et al., 2013; Kalueff et al., 2016; Konopka and Roberts, 2016) and a projection from motor cortex to the dorsal striatum is proposed for the USVs production in mice (Arriaga et al., 2012; Arriaga and Jarvis, 2013). In this model, isolation may be sensed by the pup’s somatosensory cortex leading to the production of USVs (Branchi et al., 2001; Scattoni et al., 2009; Portfors and Perkel, 2014). However it has also been reported that neonatal (P9) mice that lack most neocortex and hippocampus (*Emx1-CRE;Esco2^F/F^*) produces similar number USVs compared to WT mice (Hammerschmidt et al., 2015). This could indicate that the altered generation of USVs, observed in our study may be due to integrin deficiency in non-cortical regions as the *NEX-Cre* transgenic will also cause recombination and deletion of β1 integrin in some non-cortical areas in the midbrain and hindbrain (Belvindrah et al., 2007). Thus, our findings are consistent with prior studies showing that cortical disruptions can lead to altered USV production but cannot exclude the possible involvement of non-cortical areas.

This study adds β1 integrin to a small list of molecules where deficiency causes a transient delay in brain structural maturation (Valiente et al., 2011; Wachi et al., 2016; Bocchi et al., 2017; Rashid et al., 2017). In addition, this study shows that the structural delay is accompanied by altered behavioral maturation in USV production. These findings raise the possibility that there are genetic controls over the pace of neuronal development that may for example, define critical periods essential for normal development and if disrupted, could contribute to some forms of neural developmental delays.

## Materials and methods

### Animal

The use of all animal in this study was approved Institutional Animal Care and Use Committee (IACUC) of SUNY Upstate Medical University. For time pregnancies, the vaginal plug discovery day was considered as embryonic day 0 (E0) and birthday is defined as E21. The pan neural paxillin conditional knockout (*Nes-Cre:Pxn* ^F/F^) was generated by crossing the Nestin-Cre (Jackson laboratory stock #003771) line with our Paxillin cKO (Jackson laboratory stock #035946) and was described previously (Rashid et al., 2017). The β1 integrin conditional line has flox sites flanking exon 3, was originally generated by Dr. Elaine Fuchs (Raghavan et al., 2000) and was obtained from Jackson laboratory (B6;129- Itgb1^tm1Efu^; Jax Stock No: 004605). Postmitotic neural specific knockouts were produced using the *NEX-Cre* transgenic mice that have a NeuroD6 promoter element driving Cre recombinase expression (Schwab et al., 2000). The *NEX-Cre* transgenic line was crossed into the floxed conditional lines to delete the targeted gene from postmitotic, excitatory cortical neurons.

### PCR genotyping

Genomic DNA digested from tail snips were used for genotyping. The following primers set were used: β1 integrin; forward primer: 5^′^-cggctcaaagcagagtgtcagtc; Reverse primer: 5^′^-ccacaactttcccagttagctctc. The wildtype allele produces a band ~160 bp and the floxed allele produces a band ~280 bp. The primers for paxillin were reported previously (Rashid et al., 2017). For the *Cre* transgene (*NEX-Cre*) the forward primer 5′-gcggtctggcagtaaaaactatc and reverse primer 5′-gtgaaacagcattgctgtcactt were used which generates a ~ 100 bp band.

### Immunohistochemistry

Transcardial perfusion of adult mice was performed with Pagano solution [250 mM sucrose, 25 mM HEPES, 2.5 mM MgCl2, 2.5 mM KCl (pH 7.4)] followed 4% PFA/1X Pagano mixture (fixative) according to the previously published procedure (Rashid et al., 2017). Adult brains were fixed in 4% PFA/1X Pagano mixture for 24 h at 4°C. Embryonic and P0 brain (after cardiac perfusion with a syringe) were dropped fixed for 1-2 h at room temperature. Brains were then embedded in 10% calf-skin gelatin (Sigma Aldrich) and post fixed with 4% PFA/1X Pagano mixture for 24 h at 4°C. A Leica VT 1000S microtome (Leica Biosystems, Buffalo Grove, IL) was used to make 100 μm sections and sections were collected in PBS + 0.01% sodium azide. For total β1 integrin staining, sections were subject to antigen retrieval by heating (80°C) in citrate buffer (10 mM sodium citrate buffer, pH 8.5) for 30 min, followed by washing in PBS (Jiao et al., 1999). Also, for BrdU staining, sections were treated with 4 N HCl for 30 min for antigen retrieval at room temperature followed by neutralization with 0.5X TBE buffer (pH 8.0) for 3 × 15 min at room temperature. Sections were blocked with PBSTx (PBS + 2% BSA + 0.5% Triton-X-100) and incubated O/N at 4°C with primary antibodies diluted in PBSTx, washed three times with PBS and incubated in the appropriate secondary antibodies (diluted in PBSTx) O/N at 4°C. The following primary antibodies were used anti-Cux1 (1:50, SantaCruz, sc-13,024), anti-Tle4 (1:1000; a kind gift from Dr. Stefano Stifani, McGill University, Canada), anti-paxillin (1:200, rabbit monoclonal; clone Y113, Abcam), anti-BrdU (1,10, DSHB), activated β1 integrin (1,100, BD Bioscience, clone: 9EG7, cat# 550531), total β1 integrin (1,100, Millipore Sigma, Clone: MB1.2 Cat# MAB1997), FAK (1,100, BD Bioscience, clone: 77/FAK, Cat# 610088), Cx26 (1,50, Invitrogen), N-cadherin (1,100, SantaCruz, cat# sc-59,987). Appropriate Alexa Fluor 488-, 555- and 647-conjuagted secondary antibodies (1,500, Invitrogen) were used to detect primary antibodies. Primary antibody omitted controls (No 1° controls) were performed to rule out the possibility of non-specific secondary antibody signals. To counterstain nuclei, Hoechst 33342 (2 μg/ml, Molecular Probes) was used. Sections were mounted in mounting media [90% glycerol; 0.5% n-propyl gallate; 20 mM Tris (pH 7.4)] in glass slides under no.1.5 coverslips (VWR) and nail polish was used to seal the edges of coverslip.

### *Ex utero* electroporation

For the analyses of focal adhesion proteins, *ex utero* electroporation were performed to label migrating neurons with 0.8 μg/uL Dcx-dsRed (Wang et al., 2007) in whole hemisphere explants as described (Nichols et al., 2013). After 2 DIV, explants were dropped fixed in 4% PFA/ 1X Pagano for 30 min for subsequent immunolabeling.

### Image processing and analysis

A Zeiss LSM 780 confocal laser scanning microscope (Advanced Fluorescent Imaging Core, SUNY Upstate Medical University, NY, United States) was used for image acquisition. For high resolution images a Plan-Apochromat 40x /1.4 NA oil immersion objective was used with 2–4 times optical zoom and z-stack of 0.5 μm interslice intervals. For layer specific cellular positioning imaging, a Plan-Apochromat 10x/0.3 NA objective was used. Images were analyzed using Fiji software (Schindelin et al., 2012). For immunosignal distribution analysis, a custom macro was used to generate 20 equal bins across the cerebral wall and the mean pixel value in each bin was given as output. Cellular position analysis were performed blind to genotypes as described previously (Rashid et al., 2017). Briefly, a 50 μm wide area was cropped after orienting the image pial side up. The distance from the WM to the individual neurons were measured and it was compared to the total cortical thickness and expressed as a percentage of that total distance. The number of Hoechst+ cells in the MZ were counted in a box of constant width of 20 μm with a variable height below pial surface to the bottom of MZ. Paxillin, 9EG7, and Cx26 puncta were counted in individual z planes. The mean pixel value was measured on flatten image of equal depth of z stacks in a region of interest (ROI) of 1400μm^2^ with a height of 70 μm below pial surface and a width of 20 μm. The proximal leading process was defined as the first 10 μm measured from the soma border. Cre negative groups were considered as control (*Pxn^F/F^* or *Itgb1^F/F^*) in the EUEP experiments. Colocalization of paxillin and β1 integrin was measured using the JaCoP plugin within FIJI (Bolte and Cordelieres, 2006). Manders Colocalization Coefficient (MCCs) (Manders et al., 1993) were determined after automated Costes thresholding (Costes et al., 2004; Dunn et al., 2011).

### Maternal separation and recording of ultrasonic vocalizations

A total of 29 pups (control = 14, *NEX-Cre:Itgb1^F/F^* = 15) USVs were recorded at P4, P6, P8, P10, and P14. Individual pups were randomly isolated from the mother’s cage and transferred to a small plastic tray. Then the plastic tray was transferred into a sound-attenuating Styrofoam chamber. An UltraSoundsGate 116H Condenser Microphone (Avisoft Bioacoustics, Germany) was mounted ~23 cm directly above the pup and was connected to a PC. The Avisoft-RECORDER 4.2.24 (Avisoft Bioacoustics) was used to record the USVs for 10 min at a 250 kHz sampling rate in a 16-bit format. The number of calls and duration of each calls were recorded in a “.WAV’ file. Files were analyzed blind to genotype according to the previous publication (Scattoni et al., 2008) with slight modification. Briefly, the files were transferred to Avisoft-SASLab Pro software (Avisoft Bioacoustics) and a fast Fourier transform was conducted with the following settings 256 FFT length, 100% frame, FlatTop, and 50%-time window overlap. The spectrograms were generated with the software default settings of a 977 Hz frequency resolution and 0.512 ms time resolution. Individual calls were detected with an automated threshold and a 10 ms hold time. The number calls and the duration of each call were analyzed using the Avisoft software. Groups were compared using a Student’s *t*-test.

### Righting reflex

A righting reflex test was performed as described previously (Scattoni et al., 2008). Briefly, a pup was randomly taken from the cage and placed on its back on a solid surface so that all four paws were upward. A stopwatch was used to monitor the time required for the pup to flip over on its belly with all four paws touching the surface. A maximum of 60 s was allowed to flip over. After one trial the pup was returned to the dam to prevent hypothermia and the next pup was taken for testing. A total of three trials were made for each pup.

### Statistical analysis

For statistical analysis and graphs, SigmaPlot 11.0 (Systat Software) was used. One-way ANOVA with a Tukey’s test was used for group-wise comparison. Adobe illustrator (Adobe, San Jose, CA) was used to make the figures. For pairwise comparisons Student’s *t*-tests were performed. A *p* value of less than 0.05 was considered a significant difference. For each group, at least three animal or embryos were used. For cell position analyses, the distribution was displayed as a box and whisker plot, where the black and red lines within the box represent median and mean, respectively. The box represents the 25th to 75th percentiles, the whiskers represent 10th to 90th percentile and the circles represent outliers. Error bars reflect the standard error of the mean (s.e.m.).

## Data availability statement

The original contributions presented in the study are included in the article/Supplementary material, further inquiries can be directed to the corresponding author.

## Author contributions

MR and EO designed the experiments and wrote and edited the manuscript. MR conducted the experiments. All authors contributed to the article and approved the submitted version.

## Funding

The work was supported by the National Institute of Neurological Disorders and Stroke (NINDS Grant R01NS066071 to EO) and from the National Institute of Allergy and Infectious Disease (NIAID Grant T32AI148099 to MR).

## Conflict of interest

The authors declare that the research was conducted in the absence of any commercial or financial relationships that could be construed as a potential conflict of interest.

## Publisher’s note

All claims expressed in this article are solely those of the authors and do not necessarily represent those of their affiliated organizations, or those of the publisher, the editors and the reviewers. Any product that may be evaluated in this article, or claim that may be made by its manufacturer, is not guaranteed or endorsed by the publisher.
