# Supplementary material for: Delayed cortical development in mice with a neural specific deletion of β1 integrin
Source: Front Neurosci. 2023 May 12;17:1158419. doi: 10.3389/fnins.2023.1158419 (PMC10213249; doi:10.3389/fnins.2023.1158419)

Supplementary Figures

Supplemental Figure 1: Controls for β1 integrin antibody staining. A) A representative section of a *NEX-Cre:Itgb1*$^{F/F}$ (knockout control) section stained with anti- β1 integrin antibody. **A')** Optically zoomed image shows little immunoreactivity in the migrating neuron (asterisks) while the remaining β1 integrin reactivity may be present in the radial glia fiber (arrow). **B)** No immunoreactivity detected in the no primary controls in WT sections. **C)** Activated β1 integrin reactivity (9EG7 antibody) staining in *NEX-Cre:Itgb1*$^{F/F}$ (knockout control) section. Pial surface was outlined by a dashed white line. Scale bar: 20 µm in C; 10 µm in A'.

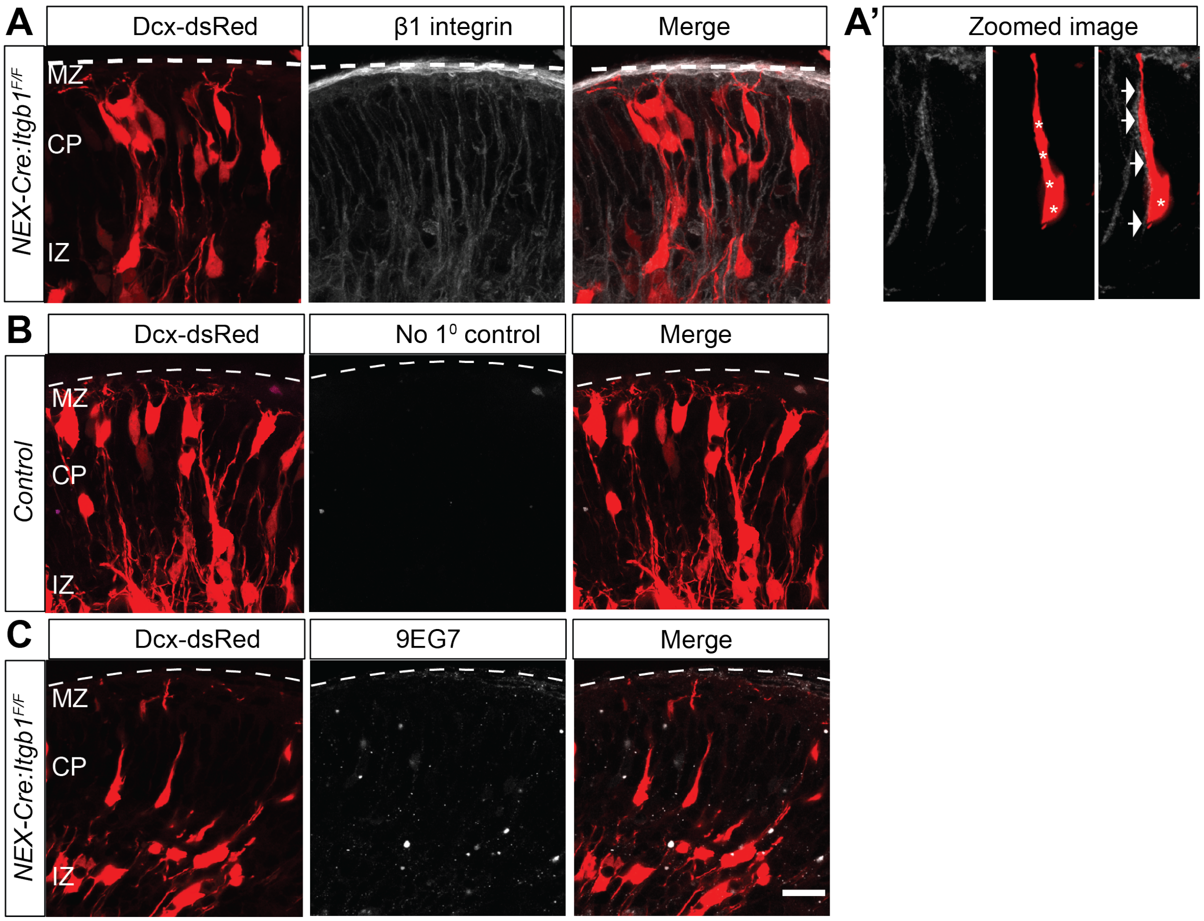
A
Dcx-dsRed
β1 integrin
Merge
A'
Zoomed image
NEX-Cre:Itgb1F/F
MZ
CP
IZ
B
Dcx-dsRed
No 1⁰ control
Merge
Control
MZ
CP
IZ
C
Dcx-dsRed
9EG7
Merge
NEX-Cre:Itgb1F/F
MZ
CP
IZ

920

921

Supplemental Figure 2: N-Cadherin expression is unaffected by the absence of paxillin or β1 integrin. A representative image of a **A)** control, **B)** *NEX-Cre:Pxn*$^{F/F}$, and **C)** *NEX-Cre:Itgb1*$^{F/F}$ section stained for N-cadherin. **D)** No difference in the mean intensity of N-cadherin was observed among the groups (n=3/group). The pial surface is outlined by a dashed white line. Scale bar: 20 µm in C. Data were analyzed by using one-way ANOVA.

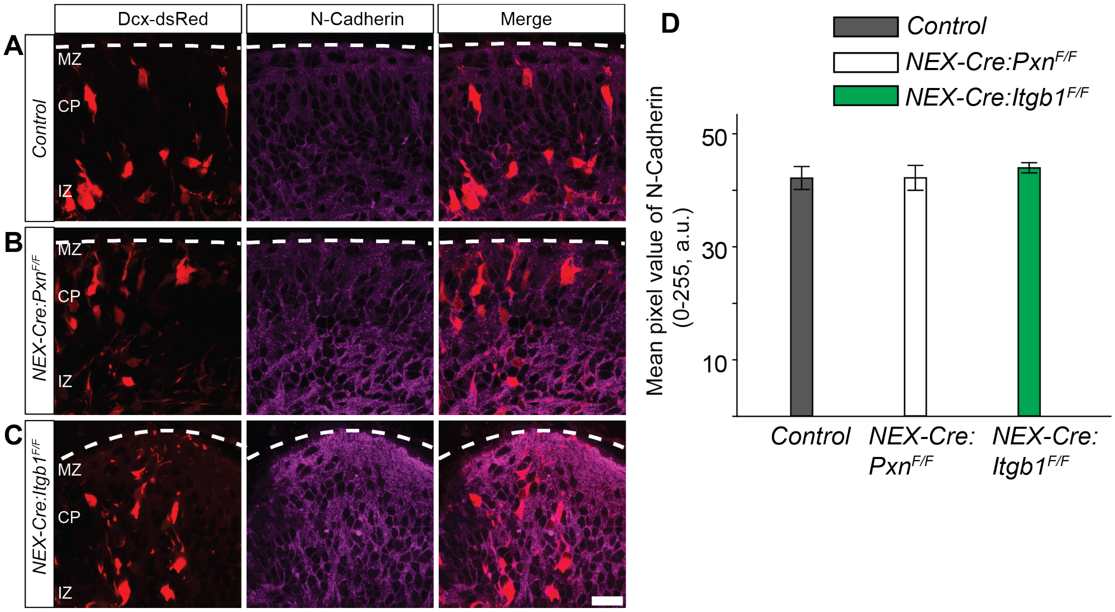
Dcx-dsRed
N-Cadherin
Merge
A
Control
MZ
CP
IZ
B
NEX-Cre:Pxn F/F
MZ
CP
IZ
C
NEX-Cre:Itgb1 F/F
MZ
CP
IZ
D
Control
NEX-Cre:Pxn F/F
NEX-Cre:Itgb1 F/F
Mean pixel value of N-Cadherin (0-255, a.u.)
50
30
10
Control
NEX-Cre: Pxn F/F
NEX-Cre: Itgb1 F/F

Supplemental Figure 3: Expression of Cx26 and FAK in paxillin and β1 integrin deficient neurons. A-C) A representative image of a **A)** control, **B)** *NEX-Cre:Pxn*$^{F/F}$, and **C)** *NEX-Cre:Itgb1*$^{F/F}$ section stained for Cx26, with corresponding zoomed image in **A'), B'),** and **C')** showing Cx26 puncta in the leading process. **D-F)** A representative image of a **D)** control, **E)** *NEX-Cre:Pxn*$^{F/F}$, and **F)** *NEX-Cre:Itgb1*$^{F/F}$ section stained for FAK which shows both puncta and nuclear expression (see Discussion). **G)** The number of Cx26 puncta was indistinguishable among the group (n=14 in cells control, n=8 cells in *NEX-Cre:Pxn*$^{F/F}$, n=10 cells in *NEX-Cre:Itgb1*$^{F/F}$*)*. **H)** No difference in the mean pixel value of FAK among the groups (n=3/groups). The pial surface is outlined by a dashed white line. Scale bar: 20 μm in F, 10 μm in C'. Data were analyzed by using one-way ANOVA.

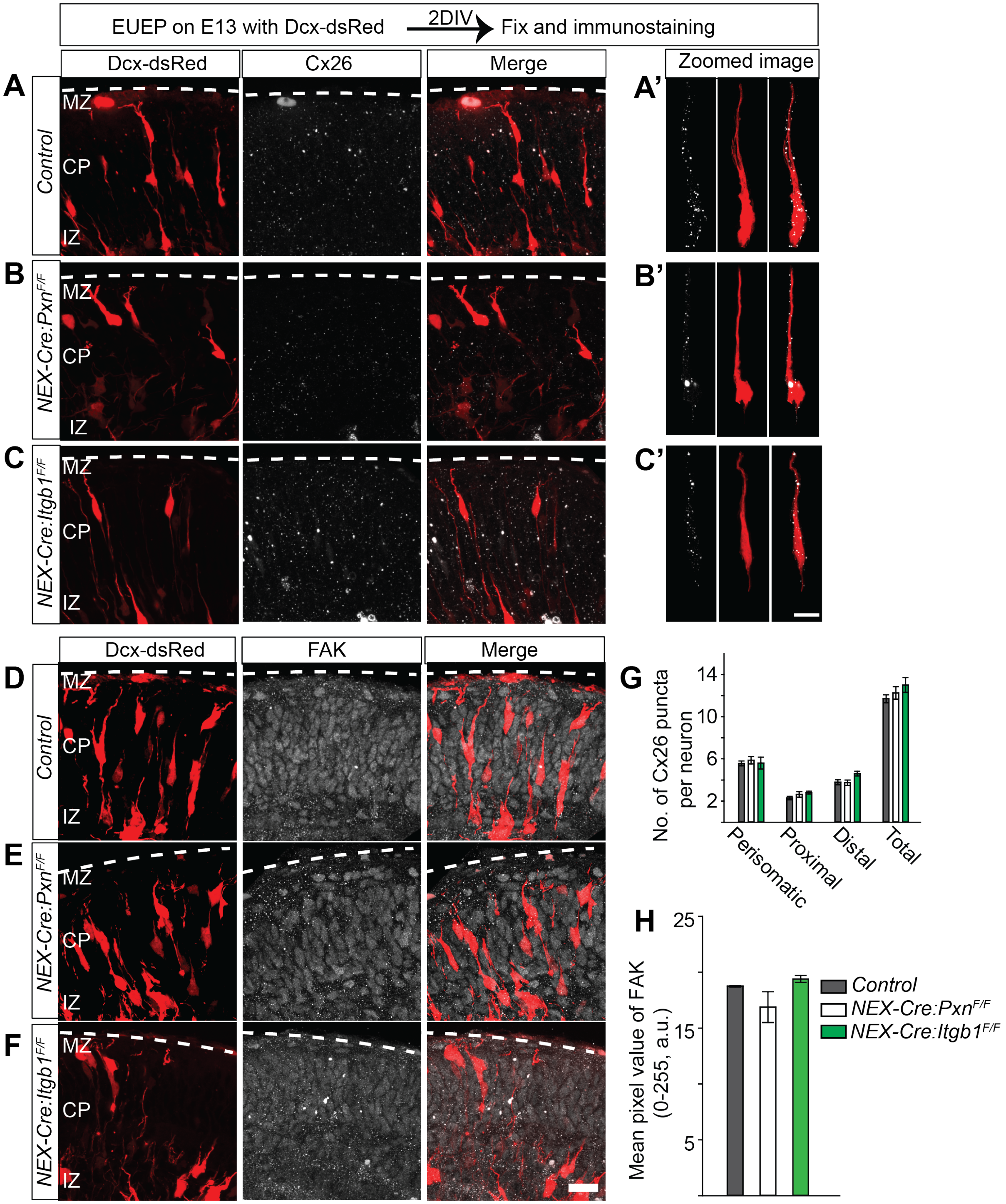
EUEP on E13 with Dcx-dsRed
2DIV
Fix and immunostaining
Dcx-dsRed
Cx26
Merge
Zoomed image
A
A'
B
B'
C
C'
Control
NEX-Cre:Pxn F/F
NEX-Cre:Itgb1 F/F
MZ
CP
IZ
Dcx-dsRed
FAK
Merge
D
E
F
G
No. of Cx26 puncta per neuron
14
10
6
2
Perisomatic
Proximal
Distal
Total
H
Mean pixel value of FAK (0-255, a.u.)
25
15
5
Control
NEX-Cre:Pxn F/F
NEX-Cre:Itgb1 F/F

Supplemental Figure 4: Expression of β1 integrin in paxillin-deficient cortical lysates. β1 integrin expression levels were analyzed from cortical lysates from E15 embryos globally lacking paxillin family member Hic-5 (Pxn$^{F/F}$ Hic-5$^{-/-}$) and specifically lacking paxillin in the developing CNS (Nestin-Cre: Pxn$^{F/F}$ Hic-5$^{-/-}$). Three replicates of each genotype are presented and quantified by densitometry. No difference in β1 integrin expression is detected in the cortical lysates despite the complete absence of paxillin family members in the developing cortical neurons (Rashid et al., 2017). The study cannot exclude that possibility that intact β1 integrin expression from the blood vessels and meningeal fibroblasts obscures smaller differences in neuronal β1 integrin expression. N=3 embryos Pxn$^{F/F}$ Hic-5$^{-/-}$ and 3 embryos from Nestin-Cre: Pxn$^{F/F}$ Hic-5$^{-/-}$. Data were analyzed using unpaired Student's t-test.

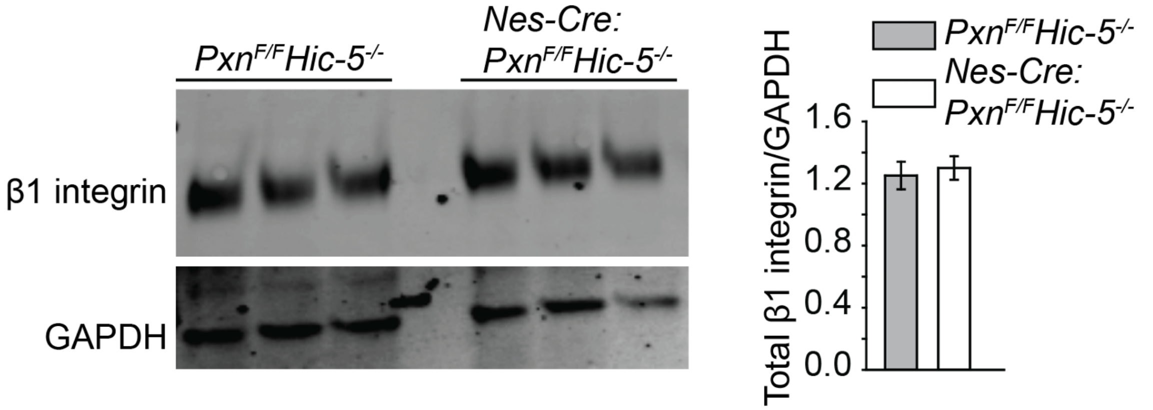

Supplement: Supplementary file 1 [file Data_Sheet_1.pdf]
